# Characterization and In Vitro Cytotoxicity Safety Screening of Fractionated Organosolv Lignin on Diverse Primary Human Cell Types Commonly Used in Tissue Engineering

**DOI:** 10.3390/biology11050696

**Published:** 2022-04-30

**Authors:** Jules A. Menima-Medzogo, Kathrin Walz, Jasmin C. Lauer, Gopakumar Sivasankarapillai, F. Robert Gleuwitz, Bernd Rolauffs, Marie-Pierre Laborie, Melanie L. Hart

**Affiliations:** 1 G.E.R.N. Center for Tissue Replacement, Regeneration & Neogenesis, Department of Orthopedics and Trauma Surgery, Faculty of Medicine, Albert-Ludwigs-University of Freiburg, Engesserstraße 4, 79108 Freiburg, Germany; menimamedzogo@outlook.de (J.A.M.-M.); walz@yahoo.de (K.W.); jasmin.lauer@uniklinik-freiburg.de (J.C.L.); bernd.rolauffs@uniklinik-freiburg.de (B.R.); 2 Faculty of Biology, University of Freiburg, Schaenzlestrasse 1, 79104 Freiburg, Germany; 3 Institute of Earth and Environmental Science, University of Freiburg, 79085 Freiburg, Germany; gopakumar.sivasankarapillai@fmf.uni-freiburg.de (G.S.); robert.gleuwitz@biomat.uni-freiburg.de (F.R.G.); marie-pierre.laborie@biomat.uni-freiburg.de (M.-P.L.); 4 Freiburg Materials Research Centre (FMF), University of Freiburg, 79104 Freiburg, Germany

**Keywords:** organosolv lignin, tissue engineering, mesenchymal stromal cells, chondrocytes, osteoblasts, fibroblasts, keratinocytes, osteoarthritis, periodontitis, gingiva, agarose

## Abstract

**Simple Summary:**

As global efforts to use eco-friendly and reusable materials increase, the use of lignin from waste biomass will continue to intensify. Lignin is an underutilized biowaste macromolecule that is gaining considerable interest in biomedical research. However, the source of lignin and the extraction process heavily influence its chemistry, which can influence a cell’s reaction to lignin. Organosolv lignin is extracted via an eco-friendly process from leftover waste material. Few studies have tested the biocompatibility of organosolv lignins with human cells. We extensively characterized fractionated organosolv lignin and performed in vitro cytotoxicity safety screening on diverse primary human cell types commonly used in tissue engineering. This is the first study to show that, at a balanced concentration, fractionated low MW beechwood-derived organosolv lignin is non-cytotoxic to highly relevant human cell types used in tissue engineering including human bone marrow-derived mesenchymal stromal cells (MSCs), chondrocytes, osteoblasts, periodontal ligament fibroblasts, gingival fibroblasts and keratinocytes. Additionally, we show that organosolv lignin can be used to fabricate cell scaffolds and that addition of lignin increased the stiffness and viscosity of the scaffolds as well as cell attachment. This suggests that organosolv lignin may be used in the generation of tissue-like biomaterial-based constructs for tissue repair.

**Abstract:**

There is limited data assessing the cytotoxic effects of organosolv lignin with cells commonly used in tissue engineering. Structural and physico-chemical characterization of fractionated organosolv lignin showed that a decrease of the molecular weight (MW) is accompanied by a less branched conformation of the phenolic biopolymer (higher S/G ratio) and an increased number of aliphatic hydroxyl functionalities. Enabling stronger polymer−solvent interactions, as proven by the Hansen solubility parameter analysis, low MW organosolv lignin (2543 g/mol) is considered to be compatible with common biomaterials. Using low MW lignin, high cell viability (70–100%) was achieved after 2 h, 24 h and 7 days using the following lignin concentrations: MSCs and osteoblasts (0.02 mg/mL), gingival fibroblasts and keratinocytes (0.02 to 0.04 mg/mL), periodontal ligament fibroblasts and chondrocytes (0.02 to 0.08 mg/mL). Cell viability was reduced at higher concentrations, indicating that high concentrations are cytotoxic. Higher cell viability was attained using 30/70 (*w*/*v*) NaOH vs. 40/60 (*w*/*v*) EtOH as the initial lignin solvent. Hydrogels containing low MW lignin (0.02 to 0.3 mg/mL) in agarose dose-dependently increased chondrocyte attachment (cell viability 84–100%) and hydrogel viscosity and stiffness to 3–11 kPa, similar to the pericellular matrix of chondrocytes. This suggests that low MW organosolv lignin may be used in many tissue engineering fields.

## 1. Introduction

Lignin ranks as the second most abundant biopolymer on earth. Despite being co-produced in massive amounts with 100 million tons produced annually as a byproduct of the pulping and paper and bioethanol production industries, it remains as an undervalued product with less than 2% of lignin utilized for high value purposes [1,2]. In recent years, concerns over environmental safety and sustainability have stimulated intense research in the use of lignin in novel fields such as use in biomedical applications, which has exponentially increased within the last 10 years [3,4].

Lignin is produced by several different plant species and the specific composition and structure of technical lignin depends on its botanical origin as well as on the extraction method applied [2]. Therefore, each type of lignin has unique physical and chemical properties, making it important to individually evaluate the different types of lignin for biocompatibility for further development of lignin in various biomedical applications [3]. Lignins extracted from biomass via the organosolv method are considered to be more environmentally friendly than other types of lignins due to the use of eco-friendly solvents and enzymes used during purification. Moreover, the organosolv process produces a high yield of lignin that is highly pure and non-sulfated and it has a good solubility, making organosolv lignin ideal for the production of pharmaceutical and biomedical applications [2]. Additionally, organosolv lignins are normally of low molecular weight, have a narrower molecular weight distribution and a low polydispersity, which tend to lead to a more homogeneous structure [2], which is highly important in producing high quality and consistent tissue engineered medicinal products (TEMPs) [5].

Lignin-based biomaterials are increasingly being used for many biomedical applications, including for drug and gene delivery, as a wound healing dressing and in tissue engineering scaffolds [3,4,6,7,8]. While cytotoxicity tests have been performed using lignin derived from biomass using techniques other than the organosolv method, to the best of our knowledge, there is only one other research group that evaluated the cytotoxic effects of organosolv lignin on two different primary human cells and showed that the addition of 1% organosolv lignin (*w*/*v*) onto bone implant coatings was non-cytotoxic to human peripheral blood mononuclear cells (PBMCs) [9] and Wharton’s jelly-derived mesenchymal stromal cells (WJ-MSCs) [10]. The few other studies that evaluated the cytotoxic effects of organosolv lignin used cancerous Caco-2 [11,12] and Saos-2 cells [13] cell lines and showed biocompatibility with these cell lines. Cytotoxicity tests of organosolv lignin with other types of primary human cells, especially those commonly used in tissue engineering, is therefore limited.

As the chemical composition of lignin can be very heterogeneous, one way to control and standardize its properties and decrease its heterogeneity is by fractionation [14,15,16]. Lower molecular weight (MW) fractionated lignin has been shown to more successfully terminate oxidative chain reactions and hence oxidative stress due to the increased abundance of phenolic hydroxyl (OH) groups present in lower MW vs. higher MW lignin fractions [15]. In addition to anti-oxidative activity, low MW lignins exhibit anti-inflammatory and anti-elastase activities [17]. Moreover, the increased phenolic groups in low MW lignins allows for increased binding of bacteria which increases bacterial cell lysis and death [3].

**Figure 1:**
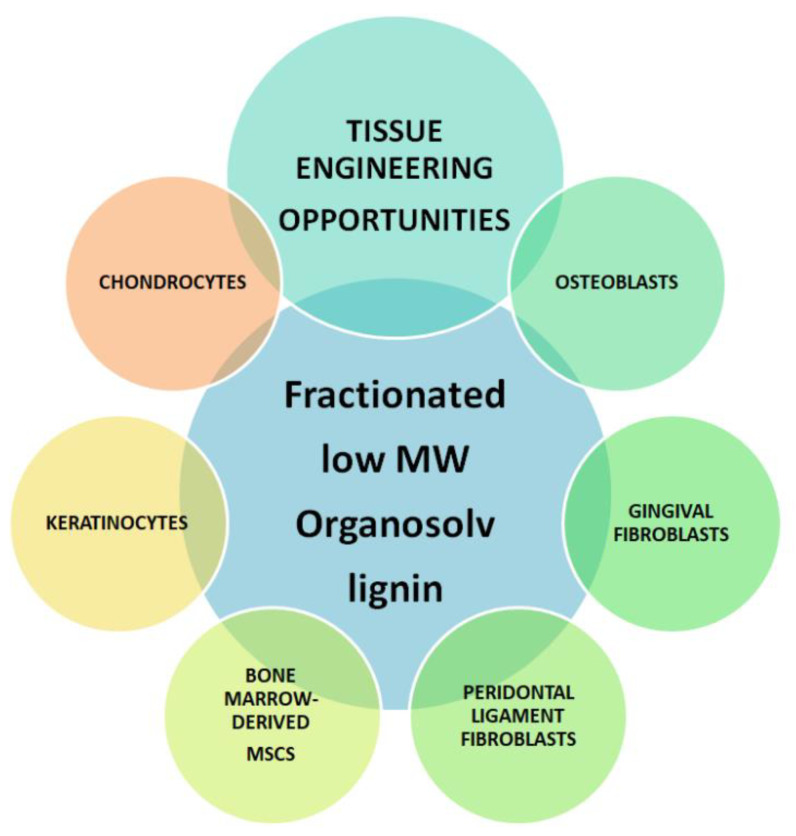
Potential of fractionated organosolv lignin in the generation of tissue-like biomaterial-based constructs for tissue repair. This can help facilitate the development of a broad spectrum of organosolv lignin-based products for future biomedical applications relevant to the fields of bone, cartilage and dental/oral tissue engineering and in wound healing applications, and, at the same time, increase the use of bio-based products.

Due to the highly diverse chemical structure of lignins, thorough characterization of each type of lignin is crucial. The aim of the present study was to extensively characterize low and high MW fractions of organosolv lignin in comparison to non-fractionated organosolv lignin and to perform cell-based in vitro cytotoxicity safety screening of fractionated low MW organosolv lignin on human primary cells commonly used in tissue engineering including human mesenchymal stromal cells (MSCs), chondrocytes, osteoblasts, periodontal ligament and gingival fibroblasts, and immortalized keratinocytes for understanding organosolv lignin’s biocompatibility and cytotoxicity. This can help open up opportunities for biomedical tissue engineering applications (Figure 1).

## 2. Materials and Methods

### 2.1. Lignin Preparation

Organosolv lignin (Batch No. KO22) derived from beechwood was kindly provided by the Fraunhofer Center for Chemical-Biotechnological Processes (CBP) (Leuna, Germany). The selected method for the fractionation of lignin at ambient temperature was based on solvent mixtures of acetic acid and water as initially reported by [18] and detailed in Gleuwitz et al. [14]. Organosolv lignin was fractionated into four different fractions using a sequential precipitation method as previously reported [14]. Of these four fractions, lignin fractions with the lowest (low MW) and the highest molecular weight (high MW) were extensively characterized for structural features and selected physico-chemical characteristics.

### 2.2. Molecular Weight Determination of Lignin Fractions

Molecular weights were determined at the Institute for Macromolecular Chemistry (University of Freiburg, Freiburg, Germany) by size exclusion chromatography using the GPC SECurity 1200 system (PSS-Polymer Standards Service, Amherst, MA, USA) at 50 °C using DMAc (+0.5% lithium bromide) as eluent and PMMA standards [14].

### 2.3. Hydroxyl Content Analysis of Lignin Fractions

Hydroxyl content analysis was performed using a quantitative ^31^P NMR procedure as published elsewhere [19]. Spectra were acquired using a Bruker 300 MHz spectrometer equipped with a quad probe at the Institute for Macromolecular Chemistry (University of Freiburg, Freiburg, Germany). An exact amount of 25−30 mg of the organosolv lignin samples was diluted in 400 μL of CDCl_3_/pyridine (1:1.6) and 150 μL of a solution of chromium(III) acetylacetonate (3.6 mg/mL) as a relaxation agent and cyclohexanol (4.0 mg/mL) as an internal standard in CDCl3/pyridine (1:1.6) was added and the solution was stirred for 5 min. 2-Chloro-4,4,5,5-tetramethyl-1,2,3-dioxaphospholane (TMDP, 70 μL) was then added, and the solution was transferred into an NMR tube for analyzing ^31^P NMR spectra with 128 scans and a delay time of 15 s.

### 2.4. Glass Transition Temperature of Lignin Fractions

The glass transition temperature (T_g_) of the powdered lignin samples were measured in sealed aluminum lids with a differential scanning calorimeter (DSC8500, PerkinElmer, Waltham, MA, USA). Samples were subjected to heat-cool-cycles in the temperature range from −20 °C to 200 °C at 20 °C/min. The T_g_ was evaluated based on the second heating scan and determined as the mid-point of the endothermic heat capacity step. A baseline measurement was performed and applied.

### 2.5. Solubility Parameters of the Lignin Fractions and Agarose

The Hansen solubility parameters (HSP) of the lignin fractions were determined according to [20]. The solubilization of the powdered lignin samples (5 mg) was tested with 47 solvents or binary solvent mixtures (1 mL) and the generated data (solubility after 48 h) was analyzed with the HSPiP software (5.0.04) and a software-integrated generic algorithm for a classical sphere fitting. The HSP of low melt agarose (Carl Roth, Karlsruhe, Germany) was evaluated based on the swelling ability of the polysaccharide (5 mg) in a set of 20 solvents or binary solvent mixtures (1 mL) after heating at 80 °C (30 min).

### 2.6. Preparation of the Low MW Fraction for In Vitro Cytotoxicity Safety Screening

For this study, the organosolv fraction with the lowest MW, denoted as low MW, was selected for cell-based in vitro cytotoxicity safety screening investigations. After fractionation, the organosolv lignin stock solution was prepared as follows. 20 mg of lignin was weighed and mixed with 200 μL ultrapure water in a 1.5 mL microcentrifuge tube using a vortexer. In a step-wise approach, 40% ethanol or 30% (*w*/*v*) sodium hydroxide was added to solubilize lignin and then heated to 85 °C while gently stirring. Two lignin stock solutions (dissolved in either a 40% ethanol or 30% (*w*/*v*) sodium hydroxide solution) containing 96.2 mg/mL low MW organosolv lignin were made and UV sterilized for 30 min. Ten serial concentrations of lignin (9.6, 4.8, 2.4, 1.2, 0.6, 0.3, 0.15, 0.08, 0.04 and 0.02 mg/mL) were produced by diluting in the cell-type-specific cell culture medium.

### 2.7. Isolation and Culture of Human-Derived Cells

Human primary cells were isolated from 3–4 different donors obtained after informed patient consent and approval by the ethics commission of the Albert-Ludwigs-University Freiburg. The following cells were isolated and characterized as previously described. Bone-marrow MSCs were obtained from the proximal femur obtained during routine hip replacement and characterized previously [21,22,23,24,25,26], while articular chondrocytes were obtained from femoral condyles during routine knee replacement [27,28,29]. Both were provided by the Clinic for Department of Orthopedics and Trauma Surgery, University Medical Center Freiburg, Germany (ethics #418/19). MSCs (passage 2, 3125 cells/cm^2^) were cultured in Dulbecco’s Modified Eagle Medium (DMEM) low glucose (Sigma Aldrich, St. Louis, MO, USA), 2 IU/mL heparin (Thermo Fisher Scientific, Waltham, MA, USA), 1% penicillin-streptomycin, 0.02 mM L-glutamine (Lonza) containing 5% human plasma (TCS Biosciences, Buckingham, UK) and 5% human pooled platelet lysate (1 × 10^8^ platelets/mL medium, Blood Donation Center, Freiburg, Germany). Chondrocytes (passage 2, 15,625 cells/cm^2^) in 1:1 (DMEM low glucose GlutaMax and F12 Nut Mix GlutaMax (Thermo Fisher Scientific, Waltham, MA, USA), 10% Fetal Bovine Serum (FBS) Superior (Sigma Aldrich, St. Louis, MO, USA), 2% Penicillin-Streptomycin (Thermo Fisher Scientific, Waltham, MA, USA), 1% Amphotericin B (Pan Biotech, Aidenbach, Germany) and 0.1 mg/mL L-Ascorbic acid phosphate magnesium salt (Sigma Aldrich St. Louis, MO, USA).

Human osteoblasts isolated from alveolar bone explants obtained during routine implant site preparation were generously provided by Dr. Brigitte Altmann, Department of Prosthetic Dentistry, University Medical Center Freiburg, Germany (ethics #411/08) [30,31,32,33]. Periodontal ligament and gingival fibroblasts that were derived from non-carious human wisdom teeth with healthy periodontium, which were extracted for orthodontic reasons, were generously provided by Dr. Susanne Proksch, Department of Operative Dentistry and Periodontology, University Medical Center Freiburg, Germany (ethics #153/15) and characterized previously [32,33,34,35]. Osteoblasts (passage 4–7, 9375 cells/cm^2^), periodontal ligament fibroblasts (passage 5–9, 9375 cells/cm^2^) and gingival fibroblasts (passage 5–7, 9375 cells/cm^2^) were cultured in DMEM low glucose containing HEPES and glutamine (Thermo Fisher Scientific, Waltham, MA, USA), 10% FBS (Sigma Aldrich, St. Louis, MO, USA), 1% glutamax (Thermo Fisher Scientific, Waltham, MA, USA) and 1% penicillin-streptomycin (Sigma Aldrich, St. Louis, MO, USA).

The parental oral gingival keratinocyte cell line, kindly provided by Prof. Pascal Tomakidi, was established by immortalization with the E6 and E7 genes of the human papilloma virus 16 (HPV-16) [36] and characterized previously [37]. Gingival keratinocytes were cultured in keratinocyte growth medium (KGM2; PromoCell, Heidelberg, Germany), including supplements and 1% penicillin-streptomycin (Sigma Aldrich, St. Louis, MO, USA).

### 2.8. Isolation and Culture of Bovine-Derived Cells

For isolation of healthy chondrocytes from bovine articular cartilage, articular cartilage discs were harvested from the patellofemoral grooves of (*n* = 2) adult 16 to 24 month old freshly slaughtered cows obtained on the day of slaughter (Emil Färber GmbH & Co. KG, Freiburg, Germany). The knee joint was opened under sterile conditions and the cartilage was removed from the femoral condyles with a scalpel. The cartilage pieces were covered with cartilage explant medium (DMEM low glucose, GlutaMAX supplement, pyruvate, Thermo Fisher Scientific, Schwerte, Germany) containing 10 mM HEPES (Pan Biotech, Aidenbach, Germany), 10% FBS superior, 2% penicillin-streptomycin, 1% amphotericin B, 0.1 mM nonessential amino acids, 0.4 mM L-proline and 0.02 mg/mL L-ascorbic acid phosphate magnesium salt) and incubated for two days at 37 °C and 5% CO_2_. Using 4 mL collagenase XI (1500 U/mL, (Sigma Aldrich, St. Louis, MO, USA), 2 mL dispase II (2.4 U/mL, Sigma Aldrich) in 6 mL chondrocyte culture medium, cells were isolated for 6 h at 37 °C and stirred with a sterile magnetic stirring bar at 250 rpm. The digest was filtered through a 100 µm cell strainer (Thermo Fisher Scientific, Waltham, MA, USA). The cell pellet was resuspended in chondrocyte culture medium and cultured in a 175 cm^²^ tissue culture flask and incubated at 37 °C and 5% CO_2_. When the cells in passage 0 were around 60 to 70% confluent, which was generally about three days later, they were split and further passaged. Passage 1 chondrocytes at a cell density of 9375 cells/cm^2^ were seeded on top of agarose or lignin-agarose hydrogels.

### 2.9. WST-1 Assay

In 96-well flat bottom plates, cells were incubated in 100 µL of the cell-specific culture medium was added and the cells were allowed to incubate overnight at 37 °C, 5% CO_2_ and 95% humidity. In vitro cell viability and proliferation testing was performed using the WST-1 kit (Roche, Basel, Switzerland) according to the manufacturer’s instructions. After cells were allowed to attach to the 96 well plates overnight, the cells were treated with serial concentrations (9.6, 4.8, 2.4, 1.2, 0.6, 0.3, 0.15, 0.08, 0.04 and 0.02 mg/mL) of the low MW fraction of lignin for 2 h, 24 h or 7 days in the cell-type-specific cell culture medium and then incubated at 37 °C, 5% CO_2_. For the 7 day cultivation period, the cell culture medium containing different lignin concentrations was changed after 96 h. *n* = 3–4 different donors were used and tested in duplicate. Washing was then carried out carefully with 100 µL warm DPBS without magnesium and calcium ions until the low MW lignin was no longer visible before measuring WST. The WST-1 reagent was diluted 1:10 (*v*/*v*) with DMEM without glucose, pyruvate, glutamine and phenol red and added to the cells. After incubation, 100 µL was removed from each well and the WST-1 reagent was added to the 96 well plate. Data was normalized using a blank of cell culture medium without cells. The cell-type-specific cell culture medium alone served as the negative control (considered as 100% of viability). Cells in cell culture medium treated with 1% (*v*/*v*) Triton-X 100 served as the positive control, which is a commonly used positive control since it results in complete cell death and prevents the WST tetrazolium salt to be intracellularly converted by dehydrogenases to the corresponding formazan product that can be measured and is in line with ISO 10993-5 guidelines for the biological evaluation of medical devices [38]. The plate was placed in the EnSight multimode plate reader (Perkin Elmer, Baesweiler, Germany) and subjected to 60 s orbital shaking before the absorbance was measured at wavelength λ = 450 nm with a reference λ = 690 nm.

### 2.10. Lignin-Agarose Hydrogels

First, 200 mg of agarose (Carl Roth, Karlsruhe, Germany) was added to a 50 mL falcon tube and mixed with 5 mL DPBS to obtain a 4% agarose solution. The agarose solution was mixed 1:1 with DPBS or a lignin solution (low MW) to obtain a 2% agarose solution and a 2% agarose solution containing 20, 75, 200 or 300 µg/mL lignin made from the NaOH or EtOH stock solution as indicated. The different agarose solutions were heated to 100 °C for 10 min in the thermomixer while shaking at 300 rpm. 142.5 µL was then pipetted into each well of a 48 well plate to obtain an agarose-lignin hydrogel with a height of approximately 1.5 mm. After solidification for 10 min at room temperature, the hydrogels were covered with 300 µL per well of chondrocyte culture medium and incubated for 16 h at 37 °C and 5% CO_2_.

### 2.11. Hydrogel Stiffness

The hydrogel stiffness was measured on day 0 and day 3 using a mechanical tester Mach-1 v500css (Biomomentum Inc. Montreal, QC, Canada), a controller from Newport, a load cell from Honeywell (1.5 N) and the software Mach-1 Motion version 4.3.1.8. A 4 mm in diameter sample was punched out of the middle of the hydrogel using a biopsy punch and then placed under the flat indenter (Ø 12.7 mm), which was connected to the load cell. The whole traverse with load cell and indenter was then moved down to find contact with the metal surface of the sample holder to normalize the mach-1 position z-axis to 0 mm (0.02 mm/s movement, contact force 1 g force [gF]) and afterwards to find contact with the samples surface (0.05 mm/s, contact force = 0.1 gF). By normalizing to 0 mm, the thickness of each specimen could then be determined for each measurement and the very low contact force avoided damaging the cylindrical specimens. An unconfined compression test was applied with an amplitude (20% of the thickness) and velocity (10% of the measured thickness). The subsequent relaxation time was set to 10 s. NaCl solution was then added to the vessel to prevent the samples from drying out. The load cell was calibrated to zero to compensate for the dead weight of the indenter and the upward force of the water and the measurement was performed. Images were recorded every 0.1 s during the measurement. The diameter of the sample was measured on the first image using ImageJ (Fiji modification version 1.52 h). This was used to calculate up to which time point a linear relationship existed. The determined time span was selected using the software *Mach-1 Analysis* version 4.1.0.17 and range the slope of the curve in gram-force/mm was obtained. The linear initial range was used to determine the Young’s modulus in kilo Pascal (kPa). For the four different agarose +/− lignin hydrogels, *n* = 3–4 hydrogels were assessed.

### 2.12. Viscoelastic Properties

The viscoelastic properties of the hydrogels were measured in duplicate at 37 °C using the UDS200 rheometer with the MP300 measurement system (Anton Paar, Graz, Austria). Complex viscosity was measured using a plate-plate configuration. The diameter of the upper round plate was 25 mm. The complex viscosity was measured with a frequency sweep in which the angular frequency was reduced from 100 to 0.1 rad/s and at a deformation (γ) of 0.1%.

### 2.13. Cell Viability of Chondrocytes on Lignin-Agarose Hydrogels

To measure cell viability, cells were stained with the fluorescent dyes Calcein AM (1 µM) and Hoechst 33,342 (1 µg/mL) (both from Thermo Fisher Scientific, Schwerte, Germany) for 1 h. Adherent cells were digitally recorded in a top-down view (Zeiss Axio Observe Z1, Oberkochen, Germany and software Fiji). Five representative images of areas within gels containing cells were chosen for analysis and used to calculate the total number of cells per well on the hydrogels. This was used to calculate the percent increase in cell number from day 0 to day 4.

### 2.14. Statistics

Statistics were performed using SigmaPlot 11.0. The statistical tests were performed using ANOVA on ranks when comparing several groups or a *t*-test when comparing two groups.

## 3. Results

### 3.1. Structural Features and Physico-Chemical Characteristics of High and Low MW Lignin Fractions

**Figure 2:**
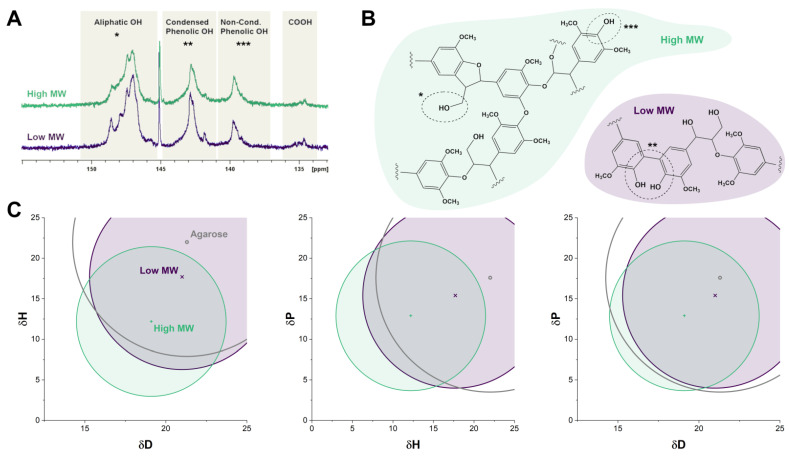
Characterization and a schematic representation of the partial molecular structure of the low and high MW fractions of organosolv lignin. (**A**) ^31^P NMR spectra of untreated and fractionated organosolv lignin in CDCl3/pyridine. The chemical shifts relative to the reaction product of TMDP with water at 132.2 ppm are assigned to the functional groups at δ = 150.0–145.5 (aliphatic-OH), 145.5–144.7 (cyclohexanol), 144.7–136.6 (phenolic-OH) and 136.6–133.6 (carboxylic acids) ppm. (**B**) Schematic illustration of the partial molecular structure of low and high MW fraction of organosolv lignin, indicating the aliphatic (*) and condensed (**) or non-condensed phenolic (***) hydroxy functionalities. (**C**) 2-D representation of HSP spheres of agarose and organosolv lignin fractions (δD = dispersion forces, δP = polar forces and δH = hydrogen bonding).

As a first step, we extensively characterized the low and high MW fractions of organosolv lignin by different techniques. In Table 1 the characteristics of the organosolv fractions with the lowest and the highest MW are summarized. Albeit still characterized by a large polydispersity, fractionation led to a distinct difference in the average molecular weight and glass transition temperatures of the extracted fractions. As shown in Table 1 and Figure 2A (estimated by ^31^P NMR analysis), the fractions have a relatively similar amount of phenolic OH groups, while a clear decrease can be observed in the number of aliphatic OH groups with increasing MW. The ratio of the lignin base units, syringyl unit (S) to guaiacyl unit (G), followed a similar trend.

Lignin is a complex polymer possessing both hydrophilicity due to its polar OH groups and hydrophobicity due to the aromatic rings. By evaluating the interaction of lignin with various solvents the compatibility with other polymers can be assessed based on the concept of interfacial adhesion [39]. Using HSP, the interactive forces responsible for compatibility between materials can be estimated. As shown in Table 1, the extracted organosolv fractions showed similar values for the parameter δ_D,_ referred to dispersive forces (van der Waals), whereas the solubility parameters associated with polarity (i.e., dipole moment δ_P_), and hydrogen bonding (δ_H_) increased with decreasing molecular weight. Next, we compared the HSP of the organosolv lignin fractions with the parameters obtained for agarose, a natural polymer commonly used in tissue engineering for cell-based scaffolds [40,41] to assess biocompatibility with another biomaterial. As shown in Figure 2C, the low MW fraction is more in accordance with the low melt agarose than the high MW lignin fraction, suggesting better compatibility.

### 3.2. In Vitro Cytotoxicity Safety Screening of Organosolv Lignin with Diverse Primary Human Cells

Because the solubility of lignin may influence the cytotoxic response, two different solvents were used to initially dissolve lignin: a 40/60 (*w*/*v*) ethanol-water vs. a 30/70 (*w*/*v*) sodium hydroxide solution. Different concentrations of lignin (ranging from 0.02 to 9.6 mg/mL), which was further diluted in DPBS, was added to the culture medium and assessed for cell viability and proliferation after 2 h, 24 h and 7 days. In vitro cytotoxicity of organosolv lignin on human-derived cells including bone marrow-derived MSCs, chondrocytes, osteoblasts, periodontal ligament fibroblasts, gingival fibroblasts and keratinocytes was measured. High cell viability was defined as ≥70% living cells.

#### 3.2.1. Bone Marrow-Derived MSCs

**Figure 3:**
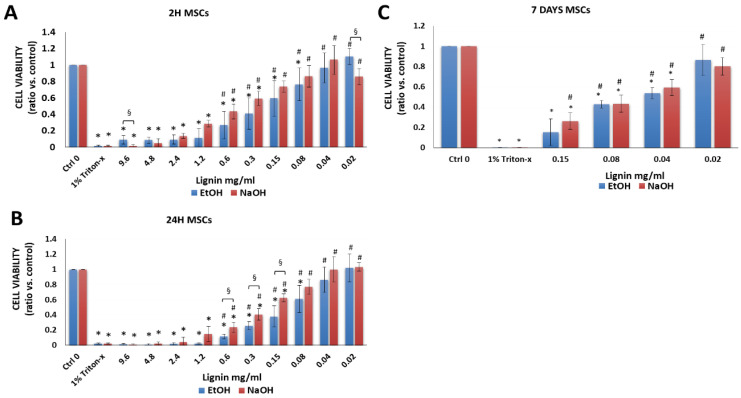
Viability of human bone-marrow derived MSCs after exposure to various concentrations of lignin for (**A**) 2 h, (**B**) 24 h and (**C**) 7 days. Data are expressed as the ratio vs. control (cell media alone without lignin). * *p* < 0.05 indicates significantly lower than negative control (media alone). ^#^ *p* < 0.05 indicates significantly higher than the positive control (1% Triton-x)—considered as 100% of cell death. ^§^ *p* < 0.05 represents a significant difference between diluted lignin that was dissolved in EtOH vs. NaOH as the initial solvent. The data are representative of the mean of *n* = 3–4 donors +/− SEM.

Multipotent bone marrow-derived MSCs have been widely explored for cell-based therapies due to their immunosuppressive, immunomodulatory, and regenerative potentials [22,26,42,43,44,45,46,47,48]. As expected, there was a significant difference between the negative control group (cells in cell culture medium alone without lignin, Ctrl 0) compared to the positive control group that was treated and, hence, lysed with 1% Triton-X-100 (Triton-x) after 2 h, 24 h or 7 days (Figure 3A–C). In comparison to cells that were cultured in media alone, after 2 and 24 h lignin was cytotoxic to cells at high lignin concentrations as demonstrated by a significant decrease in viability vs. the negative control (media alone) (Figure 3A,B). However, lower concentrations of lignin were not significantly different than cells treated with media alone using 0.02 to 0.04 mg/mL lignin (initial solvent: EtOH) and 0.02 to 0.15 mg/mL lignin (initial solvent: NaOH) after 2 and 24 h. Moreover, in comparison to the positive control (1% Triton-x treated cells), there was a significant increase in the cell viability of MSCs using 0.02 to 0.6 mg/mL lignin (EtOH or NaOH). Because the lower concentrations of lignin were more biocompatible with MSCs after 2 and 24 h, we measured the effects of long-term exposure of MSCs to lignin at these concentrations (Figure 3C). While cytotoxic effects appeared to increase with increasing exposure period, the lowest concentration of lignin (0.02 mg/mL) was biocompatible with MSCs and showed a viability of 80–100% at all time points investigated (Supplemental Figure S2A). At some concentrations, there was a significant decrease in the cell viability of MSCs when EtOH was used as the lignin solvent vs. NaOH (Figure 3A,B).

#### 3.2.2. Chondrocytes

**Figure 4:**
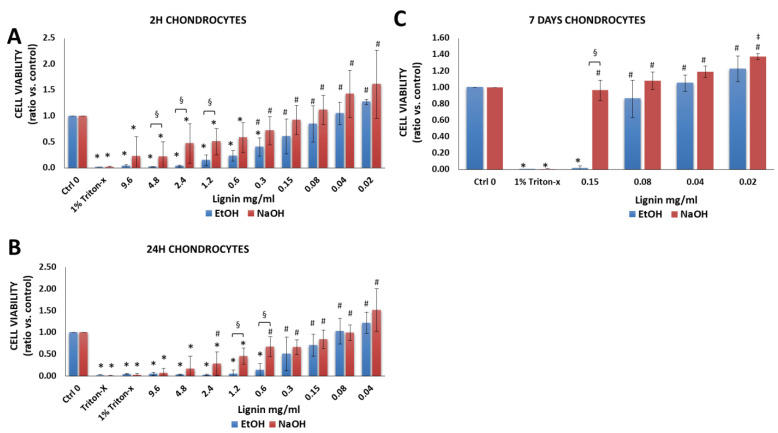
Viability of human chondrocytes after exposure to various concentrations of lignin for (**A**) 2 h, (**B**) 24 h and (**C**) 7 days. Data are expressed as the ratio vs. control (cell media alone without lignin). * *p* < 0.05 indicates significantly lower than negative control (media alone). ^#^ *p* < 0.05 indicates significantly higher than the positive control (1% Triton-x)—considered as 100% of cell death. ^§^ *p* < 0.05 represents a significant difference between diluted lignin that was dissolved in EtOH vs. NaOH as the initial solvent. The data are representative of the mean of *n* = 3–4 donors +/− SEM.

Chondrocytes are typically used to treat cartilage defects [49] and remain a large focus in developing cartilage tissue engineering strategies [29,50,51,52,53]. There was a significant decrease in the cell viability when chondrocytes were treated with 1% Triton-X-100 (Figure 4A–C) compared to chondrocytes cultured in cell medium alone without lignin (Ctrl 0). In comparison to cells that were cultured in media alone, lignin had cytotoxic effects but only at very high lignin concentrations ranging from 0.3 to 9.6 mg/mL. Lignin showed little cytotoxic effects at concentrations ranging from 0.02 to 0.08 mg/mL as the cell viability was 78–100% within this concentration range (supplemental Figure S2B). For chondrocytes, at most lignin concentrations, significantly higher cell viability was attained when NaOH was used as a solvent as opposed to EtOH (Figure 4A–C).

#### 3.2.3. Osteoblasts

**Figure 5:**
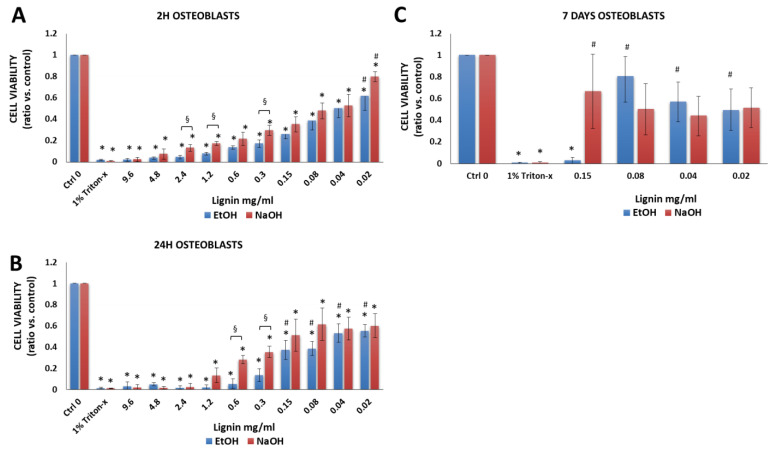
Viability of human osteoblasts after exposure to various concentrations of lignin for (**A**) 2 h, (**B**) 24 h and (**C**) 7 days. Data are expressed as the ratio vs. control (cell media alone without lignin). * *p* < 0.05 indicates significantly lower than negative control (media alone). ^#^ *p* < 0.05 indicates significantly higher than the positive control (1% Triton-x)—considered as 100% of cell death. ^§^ *p* < 0.05 represents a significant difference between diluted lignin that was dissolved in EtOH vs. NaOH as the initial solvent. The data are representative of the mean of *n* = 3–4 donors +/− SEM.

**Figure 6:**
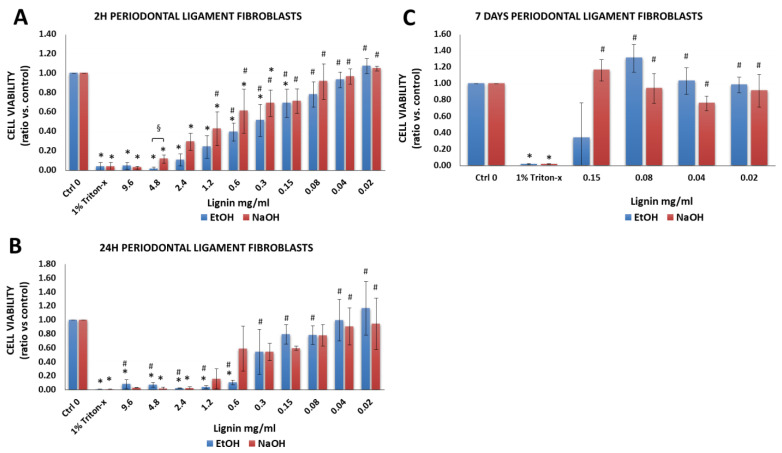
Viability of human periodontal ligament fibroblasts after exposure to various concentrations of lignin for (**A**) 2 h, (**B**) 24 h and (**C**) 7 days. Data are expressed as the ratio vs. control (cell media alone without lignin). * *p* < 0.05 indicates significantly lower than negative control (media alone). ^#^ *p* < 0.05 indicates significantly higher than the positive control (1% Triton-x)—considered as 100% of cell death. ^§^ *p* < 0.05 represents a significant difference between diluted lignin that was dissolved in EtOH vs. NaOH as the initial solvent. The data are representative of the mean of *n* = 3–4 donors +/− SEM.

Osteoblasts were chosen since they have been used bone tissue engineering [54]. Moreover, in vivo, osteoblasts are the first cells that bind to and repopulate the surface of bone implants and allow the new bone tissue to grow [55]. As expected, there was a significant difference between the negative (osteoblasts in cell culture medium without lignin, Ctrl 0) vs. positive control (Triton-x) (Figure 5A,C). However, as opposed to MSCs (Figure 3) and chondrocytes (Figure 4), lignin was not as biocompatible with osteoblasts (Figure 5). Hence, after 2 and 24 h, at all concentrations tested, there was a significant decrease in cell viability of osteoblasts vs. the negative control (media alone) (Figure 6A,B). After 2 and 24 h, a cell viability of 50–80% was achieved at the lower lignin concentration range (0.02 to 0.04 mg/mL). However, day 7 data showed that 0.02 to 0.08 mg/mL lignin resulted in 44–81% cell viability (Figure 5C, Supplemental Figure S2C). In terms of the lignin solvent, at some concentrations, significantly higher cell viability was achieved when NaOH was used as a solvent as opposed to EtOH.

#### 3.2.4. Periodontal Ligament Fibroblasts

Periodontal ligament fibroblasts were chosen due to their inherent osteogenic capacity and potential to regenerate alveolar bone [32,33,34,35,56]. Positive control group (1% Triton-X-100) treated periodontal ligament fibroblasts demonstrated a significant decrease in cell viability vs. cells cultured in cell medium without lignin (Ctrl 0) (Figure 6A–C). In comparison to cells that were cultured in media alone, lignin had cytotoxic effects but only at very high lignin concentrations ranging from 0.3 to 9.6 mg/mL. Lignin showed little cytotoxic effects (viability was between 78–100%) at concentrations ranging from 0.02 to 0.08 mg/mL (Supplementary Figure S2D). In terms of the lignin solvent effect on cell viability, with the exception of cells treated for 2 h with 5 mg/mL lignin, which showed a significant decrease in the cell viability when EtOH was used as the lignin solvent vs. NaOH (Figure 6A), there were no differences between the two groups.

#### 3.2.5. Gingival Fibroblasts

**Figure 7:**
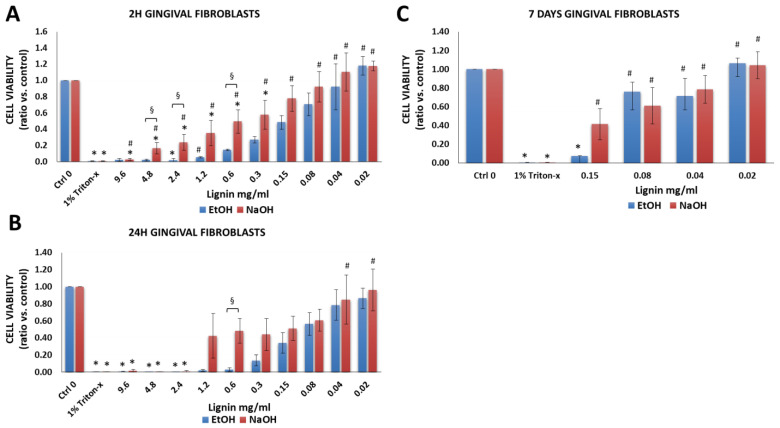
Viability of human gingival fibroblasts after exposure to various concentrations of lignin for (**A**) 2 h, (**B**) 24 h and (**C**) 7 days. Data are expressed as the ratio vs. control (cell media alone without lignin). * *p* < 0.05 indicates significantly lower than negative control (media alone). ^#^ *p* < 0.05 indicates significantly higher than the positive control (1% Triton-x)—considered as 100% of cell death. ^§^ *p* < 0.05 represents a significant difference between diluted lignin that was dissolved in EtOH vs. NaOH as the initial solvent. Blue shading indicates 70% or more of living cells. The data are representative of the mean of *n* = 3–4 donors +/− SEM.

Gingival fibroblasts were investigated based on their use in gingival and soft tissue augmentation or to treat gingival recessions [32,33,34,35,57]. There was a significant decrease in the cell viability of gingival fibroblasts that were treated with 1% Triton-X-100 (Figure 7A–C) vs. cells cultured in cell medium without lignin (Ctrl 0). In comparison to cells that were cultured in media alone, lignin had cytotoxic effects but only at very high lignin concentrations ranging from 0.3 to 9.6 mg/mL. Lignin showed little cytotoxic effects (viability was between 70–100%) at concentrations ranging from 0.02 to 0.04 mg/mL (Supplementary Figure S2E). For gingival fibroblasts, at some concentrations, significantly higher cell viability was attained when NaOH was used as a solvent as opposed to EtOH (Figure 7).

#### 3.2.6. Immortalized Human Keratinocytes

**Figure 8:**
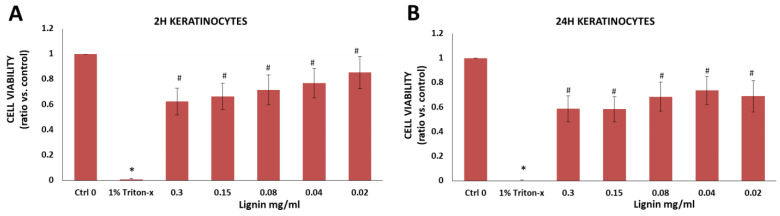
Viability of immortalized keratinocytes after exposure to various concentrations of lignin for (**A**) 2 h and (**B**) 24 h. Data are expressed as the ratio vs. control (cell media alone without lignin). * *p* < 0.05 indicates significantly lower than negative control (media alone). ^#^ *p* < 0.05 indicates significantly higher than the positive control (1% Triton-x)—considered as 100% of cell death. The data are representative of the mean of *n* = 3–4 donors +/− SEM.

Since both primary keratinocytes and immortalized keratinocytes are used to engineer human gingiva [36,37] and skin [58,59] equivalents, we also measured the effects of organosolv lignin on the immortalized keratinocyte cell line [36,37]. For keratinocytes, cell viability was only measured after 2 and 24 h and NaOH was chosen as the initial lignin solvent since all of the other cells demonstrated significantly higher cell viability when NaOH was used as a solvent vs. EtOH (Figure 3, Figure 4, Figure 5, Figure 6 and Figure 7). Similar to the other cells (Figure 3, Figure 4, Figure 5, Figure 6 and Figure 7), there was a significant difference between the negative control group (keratinocytes in cell culture medium without lignin, Ctrl 0) vs. the positive control (Triton-x) after 2 h and 24 h (Figure 8A,B). In comparison to the positive control (1% Triton-x treated cells), there was a significant increase in the cell viability of keratinocytes at all concentrations tested (0.02 to 0.3 mg/mL lignin). The cell viability was between 69–85% using 0.02 to 0.08 mg/mL lignin as indicated in blue (Supplementary Figure S2F).

### 3.3. Using Low MW Organosolv Lignin with Another Biomaterial to Fabricate Hydrogels That May Be Potentially Useful for Tissue Engineering Applications

**Figure 9:**
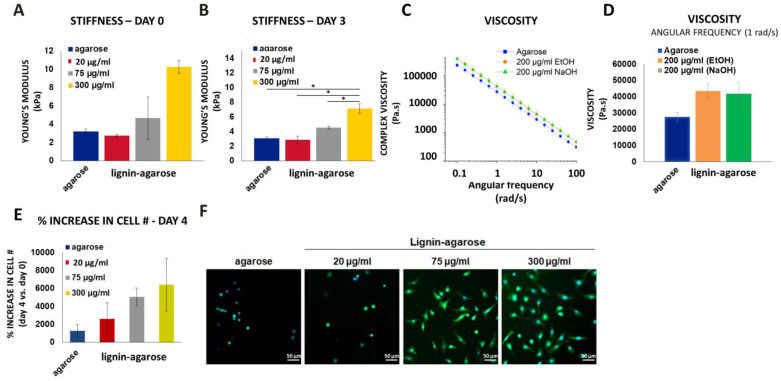
Characterization of agarose vs. lignin-agarose hydrogels. The stiffness of the hydrogels on (**A**) day 0 and (**B**) day 3. The data are representative of the mean of *n* = 3–4 +/− SEM. * *p* < 0.05 indicates a significant difference between the two groups. (**C**,**D**) Complex viscosity, as a function of angular frequency, for hydrogels at 37 °C. (**E**) The percent increase in attached chondrocytes on day 4 vs. day 0. (**F**) Fluorescent microscopic images of calcein (green) and Hoechst (nuclei, blue) stained cells on hydrogels (scale bar 50 µm). The data are representative of the mean of *n* = 6–9 +/−SEM. Hydrogels containing lignin were prepared using the low MW lignin dissolved in the 30/70 (*w*/*v*) NaOH (**A**,**F**) or 40/60 (*w*/*v*) EtOH (**C**,**D**) solvent.

As the next step, we made lignin-agarose hydrogel composites. To determine if lignin in an agarose hydrogel modulated the stiffness of agarose hydrogels, 2% agarose hydrogels were prepared using 20, 75 or 300 µg/mL lignin and compared to hydrogels containing agarose alone. On day 0, there was a dose-dependent trend showing that the addition of lignin to hydrogels increased the Young’s modulus compared to agarose hydrogels alone (Figure 9A). By day 3, this trend became apparent with agarose hydrogels containing 300 µg/mL lignin having a significantly higher Young’s modulus vs. agarose hydrogels alone or hydrogels containing 20 or 75 µg/mL lignin (Figure 9B). This is likely due to the increase in viscosity when using higher concentrations of lignin in the lignin-agarose hydrogels vs. agarose hydrogels alone (Figure 9C,D). When chondrocytes were seeded onto agarose and lignin-agarose hydrogels, the addition of lignin to agarose supported cell adhesion but also led to a more spread morphology, and dose-dependently increased the percentage of bound cells (Figure 9E,F).

## 4. Discussion

Structural and physico-chemical characterization of high and low MW lignin fractions showed that, overall, the low MW fraction of organosolv lignin was found to be intrinsically different than the high MW fraction in regards to homogeneity, hydrogen bonding capacities and physico-chemical properties. The low MW fraction, with its highest S/G ratio, is considered to have a less branched molecular structure compared to the high MW fraction as supported by the depression of T_g_ [60,61,62]. Based on this difference in conformation and the increased number of aliphatic hydroxyl functional groups, intermolecular interactions are promoted for low MW lignin, enabling stronger polymer−solvent interactions compared to the high MW fraction. Hereby, the effect of increased hydrogen bonding should outweigh the hydrophobic interactions due to the higher amount of condensed phenolic structures in the low MW fraction [63]. Due to these features, it is expected that the low MW lignin fraction is more compatible with biomaterials, as we showed with agarose, due to an optimum chain intermingling and availability of its functional sites in the hydrogel.

There is limited data on the cytotoxic effects of organosolv lignin with cell types commonly used in tissue engineering. In primary human cells organosolv lignin, which was extracted from a different botanical origin compared to the present study, has only been shown to be biocompatible with human PBMCs [9] and WJ-MSCs [10] as well as two immortalized cell lines (Caco-2 [11,12] and Saos-2 cells [13]). We extend these studies and show for the first time that, at a balanced lignin concentration, the low MW fraction of beechwood-derived organosolv lignin is non-cytotoxic to highly relevant human cell types used in tissue engineering.

We present data at several time points and over an extended period of time, showing good biocompatibility up to 7 days. This shows that long-term treatment using low concentrations of organosolv lignin is not detrimental. Using a wide variety of human cell types used in tissue engineering, we show that a high cell viability (defined as 70–100% viability) can be achieved for up to 7 days using the following concentrations of soluble lignin: MSCs (0.02 mg/mL), chondrocytes (0.02 to 0.08 mg/mL), osteoblasts (0.02 mg/mL or less), periodontal ligament fibroblasts (0.02 to 0.08 mg/mL), gingival fibroblasts (0.02 to 0.04 mg/mL) and keratinocytes (0.02 to 0.04 mg/mL). High cell viability (84–100%) was also achieved for up to 4 days when chondrocytes were cultured on lignin (0.02 to 0.3 mg/mL)-2% agarose hydrogels. Moreover, we show that a 40/60 (*w*/*v*) ethanol-water and a 30/70 (*w*/*v*) sodium hydroxide-water solution are efficient solvents for initially dissolving the low MW isolated organosolv lignin fraction but that higher cell viability was attained when NaOH was used as a solvent compared to EtOH.

Many substances or materials can be cytotoxic at high concentrations. We tested a broad range of organosolv lignin concentrations, including very high concentrations. Cell viability was significantly reduced compared to control for all of the cells tested in the present study when using high organosolv lignin concentrations (0.3 to 9.6 mg/mL). Similar to our study, other studies have also shown that high doses of lignin, derived from other biomass sources or isolated via different methods, also inhibited cell proliferation and were cytotoxic to cells including keratinocytes and MSCs [64,65,66,67]. This indicates that high concentrations of organosolv lignin are cytotoxic but the lower concentrations are non-cytotoxic when lignin is used alone or when it is incorporated into another biomaterial.

In this study we additionally show that organosolv lignin can be used to fabricate cell scaffolds and that addition of lignin increased the stiffness and viscosity of the scaffolds as well as cell attachment. As a representative example, we chose to combine lignin with agarose because it is a natural polymer commonly used in tissue engineering applications due to its similarity to the extracellular matrix of multiple tissues types, its tunable properties, and inert characteristics that does not elicit a substantial immune response, making it an ideal base material for composite cell-based scaffolds [40,41]. Moreover, agarose is a polysaccharide that resembles the extracellular matrix (ECM) of cartilage and it has been widely used in combination with chondrocytes at a concentration of 2% [40,68,69,70,71]. In the present study, 2% agarose was combined with 20, 75 or 300 µg/mL organosolv lignin. While the lower concentrations of soluble lignin (20 µg/mL and 75 µg/mL) in chondrocyte medium were non-cytotoxic and biocompatible with chondrocytes (84–100% cell viability up to 7 days when NaOH was used as the solvent), the higher concentration of soluble lignin (300 µg/mL) in chondrocyte medium had a slightly cytotoxic effect when chondrocytes were cultured with soluble lignin, with a 72% and 67% viability after 2 and 24 h, respectively, but a lower (25%) viability after 7 days (Figure 5). Despite this effect, we chose to use the higher lignin concentration in the hydrogels since lignin is distributed throughout the hydrogel and showed that the addition of 300 µg/mL of lignin to agarose hydrogels led to the best cell attachment and increased the biocompatibility of chondrocytes cultured on lignin-agarose scaffolds (Figure 9. This may be due to the dose-dependent increase in hydrogel viscosity and stiffness that correlated with increased concentrations of lignin in the hydrogels. This confirms the hypothesis that lignin is capable of increasing the stiffness of agarose hydrogels and increasing adherence of chondrocytes to agarose hydrogels. Importantly, we found that the stiffness of the lignin-agarose hydrogels was between 3–11 kPa. This stiffness falls within the range of the pericellular matrix (PCM) that immediately surrounds chondrocytes [52,72] and is similar to 2% agarose hydrogels that led to ECM deposition and integration of articular cartilage in chondrocyte-containing agarose hydrogels [69,70,71]. The additional benefits of incorporating lignin into agarose or other cell hydrogel scaffolds for any cell type include lignin’s anti-oxidant effects [15,17,64,66,67], its ability to regulate inflammation [17,65,73] and promote bacterial cell death [3] and prevent microbial colonization [9,10]. These positive lignin-mediated biomodulatory effects may greatly enhance tissue engineering approaches.

Two studies have already used organosolv lignin as a surface treatment strategy to help mitigate the risk of implant-related bone infection [9,10], which is a major problem in orthopedic and trauma-related surgery and although rare, is also associated with dental implants [74]. These studies showed that coating titanium bone implants with 1% (*w*/*v*) organosolv lignin increased the antimicrobial efficacy against *Staphylococcus aureus, Pseudomonas aeruginosa* and *Candida famata* [9,10], which can colonize the skin and mucous membranes and cause prosthesis-related infections and inflammatory destruction of the joint and bone [75]. While these important studies showed good biocompatibility with human PBMCs [9] and Wharton’s Jelly-derived MSCs, including growth and adherence of the MSCs [10], these studies did not investigate biocompatibility with osteoblasts. Osteoblasts are the initial cell to bind, spread, proliferate and differentiate on the implant surface, which leads to new bone growth [55]. Our results show that lower concentrations of organosolv lignin (0.02 to 0.08 mg/mL) are biocompatible with osteoblasts (44–80% cell viability depending on the day and solvent used). We previously showed that, by analyzing different surface modifications, implant surface topography controls osteoblast cell morphology and subsequent cell proliferation [31]. Moreover, we showed that surface texture and surface enlargement are more effective than surface roughness or wettability in controlling osteoblast morphogenesis and proliferation on implant biomaterials. This suggests that, under the appropriate circumstances and concentrations, organosolv lignin may be beneficial as an implant coating material but that future studies need to determine how organosolv-lignin-coated implants influence osteogenic cell morphology, cell proliferation and osteointegration.

As global efforts continue to use eco-friendly materials and use of reusable materials increases, lignin-based biomedical research will continue to intensify [3,4]. The biocompatibility of organosolv lignin with bone-marrow derived MSCs, chondrocytes, osteoblasts, periodontal ligament and gingival fibroblasts as well as keratinocytes suggest that it can be used in many tissue engineering fields. This data highlights the versatile potential of organosolv lignin in biomedical applications and shows that organosolv lignin may be applied to biomedical applications relevant to the fields of bone, cartilage and dental/oral tissue engineering and in wound healing applications. This may facilitate the development of a broad spectrum of organosolv lignin-based products for future biomedical applications and at the same time increase the use of bio-based products.

## Figures and Tables

**Table 1:** Summary of the characterization of organosolv lignin fractions.

	SEC Data		^31^P NMR Data			DSC Data	Hansen Solubility Parameters		
Fraction	MW (g/mol)	Polydispersity Index (Mw/Mn)	Aliphatic OH (mmol/g)	Phenolic OH (mmol/g)	S/G Ratio	Tg/°C	δ_D_(MPa^1/2^)	δ_P_(MPa^1/2^)	δ_H_(MPa^1/2^)
Raw lignin	3723	3.2	2.43 *	2.20 *	2.0 *				
Low MW fraction	2543	2.2	2.72	1.91	2.62	123	21.0	15.4	17.7
High MW fraction	6025	3.1	1.95	1.87	2.10	174	19.1	12.9	12.2

Abbreviations: SEC (size-exclusion chromatography), NMR (nuclear magnetic resonance), DSC (differential scanning calorimetry), S/G (syringyl to guaiacyl), MW (molecular weight), Mn (number averaged MW), T_g_ (glass transition temperature), δ_D,_ (dispersive forces), δ_P_ (dipole moment) and δ_H_ (hydrogen bonding). * Data from our previous work [14]. DSC thermograms can be found in supplemental Figure S1.

## Data Availability

The datasets used and/or analyzed during the current study are available from one of the corresponding authors upon reasonable request.

## Supplementary Materials

The following supporting information can be downloaded at: https://www.mdpi.com/article/10.3390/biology11050696/s1, Figure S1: DSC thermogram of organosolv lignin fractions (second heating cycle) under nitrogen atmosphere at a heating rate of 20 K/min.); and Figure S2: Percent cell viability compared to untreated cells.
