# Supplementary material for: Characterization and In Vitro Cytotoxicity Safety Screening of Fractionated Organosolv Lignin on Diverse Primary Human Cell Types Commonly Used in Tissue Engineering
Source: Biology (Basel). 2022 Apr 30;11(5):696. doi: 10.3390/biology11050696 (PMC9139013; doi:10.3390/biology11050696)
Supplement: Supplementary file 1 [file biology-11-00696-s001.zip › biology-1701508-supplementary.pdf]

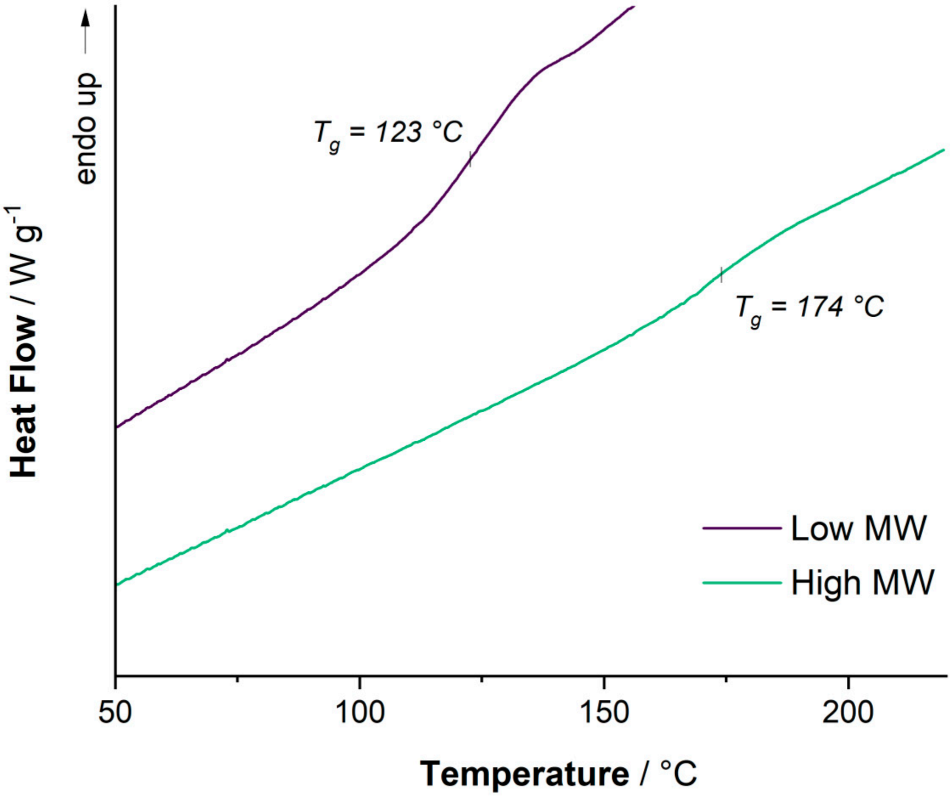

Supplemental Figure S1. DSC thermogram of organosolv lignin fractions (second heating cycle) under nitrogen atmosphere at a heating rate of 20 K/min. A baseline measurement was performed and applied.

A

Cell	Lignin Conc (mg/ml)	Cell viability (% of control)					
		2 h		24 h		7 days	
		EtOH	NaOH	EtOH	NaOH	EtOH	NaOH
MSCs	0	100	100	100	100	100	100
MSCs	9.6	9	1	1	0		
MSCs	4.8	8	5	1	2		
MSCs	2.4	9	14	2	4		
MSCs	1.2	11	29	2	15		
MSCs	0.6	27	44	11	24		
MSCs	0.3	41	59	26	41		
MSCs	0.15	59	74	38	62	15	26
MSCs	0.08	76	86	61	77	43	43
MSCs	0.04	97	100	86	100	54	59
MSCs	0.02	100	86	100	100	86	80

B

Cell	Lignin Conc (mg/ml)	Cell viability (% of control)					
		2 h		24 h		7 days	
		EtOH	NaOH	EtOH	NaOH	EtOH	NaOH
Chondrocytes	0	100	100	100	100	100	100
Chondrocytes	9.6	4	22	4	3	3	1
Chondrocytes	4.8	2	21	5	6	3	0
Chondrocytes	2.4	4	47	3	17	4	0
Chondrocytes	1.2	15	51	3	28	2	0
Chondrocytes	0.6	23	58	5	46	2	1
Chondrocytes	0.3	40	72	14	67	0	25
Chondrocytes	0.15	61	92	51	66	2	97
Chondrocytes	0.08	85	100	71	84	86	100
Chondrocytes	0.04	100	100	100	99	100	100
Chondrocytes	0.02	100	100	100	100	100	100

C

Cell	Lignin Conc (mg/ml)	Cell viability (% of control)					
		2 h		24 h		7 days	
		EtOH	NaOH	EtOH	NaOH	EtOH	NaOH
Osteoblasts	0	100	100	100	100	100	100
Osteoblasts	9.6	2	2	3	2	2	1
Osteoblasts	4.8	4	8	5	1	3	2
Osteoblasts	2.4	5	13	2	2	1	1
Osteoblasts	1.2	8	17	2	13	2	2
Osteoblasts	0.6	14	22	5	28	1	5
Osteoblasts	0.3	17	30	14	35	0	41
Osteoblasts	0.15	26	35	37	51	3	67
Osteoblasts	0.08	38	48	38	62	81	50
Osteoblasts	0.04	50	53	53	57	57	44
Osteoblasts	0.02	62	80	55	60	49	52

D

Cell	Lignin Conc (mg/ml)	Cell viability (% of control)					
		2 h		24 h		7 days	
		EtOH	NaOH	EtOH	NaOH	EtOH	NaOH
PDL fibro	0	100	100	100	100	100	100
PDL fibro	9.6	5	3	8	3	4	1
PDL fibro	4.8	2	12	7	2	5	2
PDL fibro	2.4	11	30	2	2	4	2
PDL fibro	1.2	24	43	4	16	3	2
PDL fibro	0.6	39	61	10	59	0	0
PDL fibro	0.3	52	69	54	54	0	66
PDL fibro	0.15	69	71	79	59	34	100
PDL fibro	0.08	78	92	78	78	100	94
PDL fibro	0.04	93	97	100	91	100	76
PDL fibro	0.02	100	100	100	94	98	91

E

Cell	Lignin Conc (mg/ml)	Cell viability (% of control)					
		2 h		24 h		7 days	
		EtOH	NaOH	EtOH	NaOH	EtOH	NaOH
**Gingival fibro**	0	100	100	100	100	100	100
**Gingival fibro**	9.6	2	2	0	2	1	1
**Gingival fibro**	4.8	2	17	0	0	0	0
**Gingival fibro**	2.4	2	24	0	1	1	1
**Gingival fibro**	1.2	5	35	2	42	0	1
**Gingival fibro**	0.6	14	49	2	48	0	7
**Gingival fibro**	0.3	27	58	13	44	0	47
**Gingival fibro**	0.15	48	78	34	51	7	41
**Gingival fibro**	0.08	71	92	56	61	76	61
**Gingival fibro**	0.04	92	100	79	85	71	79
**Gingival fibro**	0.02	100	100	87	96	100	100

F

Cell	Lignin Conc (mg/ml)		
		2 h	24 h
		NaOH	NaOH
**Keratinocytes**	0	100	100
**Keratinocytes**	0.3	62	59
**Keratinocytes**	0.15	66	58
**Keratinocytes**	0.08	72	69
**Keratinocytes**	0.04	77	74
**Keratinocytes**	0.02	85	70

Supplemental Figure S2. Percent cell viability compared to untreated cells. A) MSCs, B) chondrocytes, C) osteoblasts, D) periodontal ligament fibroblasts, E) gingival fibroblasts, and F) keratinocytes. Blue shading indicates 70% or more of living cells compared to the negative control (considered as 100% viability). The data are representative of the mean of n=3-4 donors +/- SEM.
